# Patterns of alternative splicing vary between species during heat stress

**DOI:** 10.1093/aobpla/ply013

**Published:** 2018-02-21

**Authors:** Sumetha Kannan, Gillian Halter, Tanya Renner, Elizabeth R Waters

**Affiliations:** 1 Department of Biology, San Diego State University, Campanile Drive, San Diego, CA, USA; 2 Department of Entomology, Pennsylvania State University, University Park, PA, USA

**Keywords:** Alternative splicing, *Arabidopsis thaliana*, *Boechera depauperata*, differential gene expression, heat stress, RNA-seq

## Abstract

Plants have evolved a variety of mechanisms to respond and adapt to abiotic stress. High temperature stress induces the heat shock response. During the heat shock response a large number of genes are up-regulated, many of which code for chaperone proteins that prevent irreversible protein aggregation and cell death. However, it is clear that heat shock is not the only mechanism involved in the plant heat stress response. Alternative splicing (AS) is also important during heat stress since this post-transcriptional regulatory mechanism can produce significant transcriptome and proteome variation. In this study, we examine AS during heat stress in the model species *Arabidopsis thaliana* and in the highly thermotolerant native California mustard *Boechera depauperata*. Analyses of AS during heat stress revealed that while a significant number of genes undergo AS and are differentially expressed (DE) during heat stress, some undergo both AS and DE. Analysis of the functional categories of genes undergoing AS indicated that enrichment patterns are different in the two species. Categories enriched in *B. depauperata* included light response genes and numerous abiotic stress response genes. Categories enriched in *A. thaliana*, but not in *B. depauperata*, included RNA processing and nucleotide binding. We conclude that AS and DE are largely independent responses to heat stress. Furthermore, this study reveals significant differences in the AS response to heat stress in the two related mustard species. This indicates AS responses to heat stress are species-specific. Future studies will explore the role of AS of specific genes in organismal thermotolerance.

## Teaser

Alternative splicing (AS) can generate significant protein variation from a single gene. While the basic process of AS has been known for some time, little is known about how AS varies across species. In addition, there are many unanswered questions about the role of AS in plant responses to abiotic stress. In this study of the model plant species, *Arabidopsis thaliana*, and a native California mustard, *Boechera depauperata*, researchers report that the genes that undergo AS are distinct in each species. This provides evidence that AS plays an important and species-specific role in the plant stress response.

## Introduction

Large temperature change can lead to an array of physiological and biochemical responses in plants that significantly affect plant growth and development. Under extreme conditions they may lead to reductions in plant growth, which in turn can drastically depress crop yields (Wahid *et al.* 2007). However, some responses help to minimize the damage. A well-known example is the heat shock response. This is a highly conserved reaction to elevated temperatures during which heat shock transcription factors (HSFs) are induced and heat shock proteins are produced (Nover *et al.* 1996; Swindell *et al.* 2007; Scharf *et al.* 2012; Qu *et al.* 2013). There have been several recent studies on the complexity of the heat stress response (Larkindale *et al.* 2005; Larkindale and Vierling 2008; Mittler *et al.* 2012) and there is much still to be learned about the mechanisms beyond the up-regulation of the much-studied heat shock proteins.

Alternative splicing (AS) is a common phenomenon in eukaryotes (Syed *et al.* 2012; Kornblihtt *et al.* 2013). It results in two or more processed mRNAs being generated from the same precursor mRNA. This process results in a diversity of mRNAs from the same gene thereby generating variant proteins. Common AS events are exon skipping (ES), intron retention (IR) and alternative 5' or 3' splicing events. While AS has been known for some time, the advent of deep sequencing RNA-seq technology has enabled more robust analyses of AS in a wide variety of plants, including the moss, *Physcomitrella patens* (Chang *et al.* 2014); and in flowering plants such as *Citrullus lanatus* (watermelon) (Saminathan *et al.* 2015) and *Triticum aestivum* (wheat) (Terashima and Takumi 2009). Recent studies have examined the frequency of AS in a variety of plant species and under different stress or growth conditions (Lopez 1998; Zhou *et al.* 2011; Ding *et al.* 2014; Kwon *et al.* 2014; Shikata *et al.* 2014). Alternative splicing has been found to be altered by red and far-red light (Shikata *et al.* 2014), changes in the circadian clock (Kwon *et al.* 2014) and to salt stress (Ding *et al.* 2014). In the moss *P. patens*, even mild heat stress can induce AS in certain functional gene classes (Chang *et al.* 2014). While it is clear from the work cited here that AS is common in plants, much is still to be learned about the process and its role in plant stress responses.

We have examined the basal and acquired heat stress response in the model plant *Arabidopsis thaliana* and in the wild plant *Boechera depauperata*. *Boechera* (Brassicaceae) is a large genus native to North America. Its species live in a variety of habitats from high elevation mountains—to coastal regions and deserts (Alexander *et al.* 2013). The combination of being closely related to *A. thaliana* and having undergone natural selection under varying climatic conditions provides a useful system with which to examine stress tolerance (Gallas and Waters 2015; Halter *et al.* 2017). To study AS and its role in heat stress, we compared AS in heat-stressed *A. thaliana* and *B. depauperata*. Previous studies have reported that *B. depauperata* can maintain photosynthesis and organismal thermotolerance under stress conditions that are two extreme for *A. thaliana* to tolerate, and that this tolerance is not the result of higher levels of heat shock protein expression (Gallas and Waters 2015; Halter *et al.* 2017). To further understand thermotolerance in *B. depauperata*, we examined AS patterns during heat stress. We found patterns of AS during heat stress in *A*. *thaliana* that were distinct from those of *B. depauperata*. Both species underwent AS but the most common splicing events varied between the two species, and between basal and acquired heat treatment. In addition, the functional classes of genes undergoing AS events differed between the two species. Finally, we found that in both species, the genes undergoing AS and that are differentially expressed were largely non-overlapping.

## Methods

Plants were grown, and basal and acquired heat stress experiments were conducted as described in (Larkindale and Vierling 2008; Gallas and Waters 2015; Halter *et al.* 2017). Basal and acquired heat stress experiments were conducted. In basal heat stress (B/HS) plants are moved directly from control (22 °C) conditions to a 3-h heat stress at 38 °C. In acquired heat stress (A/HS) plants are exposed to a 1-h 38 °C heat treatment, followed by a 1-h recovery at 22 °C, and then a 3-h heat stress at 43 °C.

Total RNA was isolated using AMBION RNAeasy isolation kits. The quality of RNA was examined using a BioAnalyzer. The RNA-seq libraries were sequenced using Illumina TruSeq according to standard protocol. Two biological replicates were independently prepared throughout the processes, from the induction of seed germination to the preparation of RNA-seq libraries. For the RNA-seq libraries a total of 4-GB paired-end reads were determined using a HighSeq Illumina instrument. The reads with either low-quality scores or adapters were partially trimmed by fastx toolkit. The raw sequencing reads can be found at the NCBI GEO database with the following accession number GSE107820.

The trimmed sequences were mapped to the annotated genes in the *A. thaliana* genome (TAIR10) using TopHat (Trapnell *et al.* 2009) or the *B. depauperata* transcriptome or *B. stricta* genome (described below). Each RNA-seq sample had high sequence coverage against 32398 annotated genes in the TAIR10 release produced by The *Arabidopsis* Information Resource (TAIR, www.arabidopsis.org). In this analysis, 82917 splice junctions (SJs) were identified in control *A. thaliana* tissue. This is consistent with published (Kwon *et al.* 2014) and publically available information (TAIR, www.arabidopsis.org). This indicates that our methodology for identifying SJs is robust.

Genome sequencing for *B. depauperata* is currently unavailable. A heat stress and control transcriptome for *B. depauperata* was therefore constructed from independent heat stress and control RNA-seq libraries and assembled using Trinity (Haas *et al.* 2013) and was annotated using annocript (Musacchia *et al.* 2015). Since *B. depauperata* is a polyploid, we disentangled the homologs using homeosplitter (Ranwez *et al.* 2013). When we used the *B. depauperata* transcriptome as reference, ~100185 junctions were identified. *Boechera stricta* is a close relative of *B. depauperata* for which a complete genome (*JGI, Phytozyome, DOE*) is available. When this was used as a reference genome, 98153 junctions were identified. We compared the junctions obtained by both methods and found that ~95 % of the junctions were identical. Hence, for the further analysis we used the *B. stricta* genome to identify different splicing events. The SJs were identified using TopHat (Trapnell *et al.* 2009). DiffSplice (Hu *et al.* 2013) was used to identify AS events. DESeq2 (Love *et al.* 2014) was used to identify the DE of genes. Finally, Integrated Genome Viewer (IGV) was used to visualize the AS events (Robinson *et al.* 2011). The *P*-values for DE were calculated using a binomial test for differences between the base means of two conditions. These *P*-values were then adjusted for multiple test correction using Benjamini–Hochberg algorithm to control the false discovery rate (FDR). We considered genes differentially expressed between two groups of samples when the DESeq2 analysis resulted in an adjusted *P*-value of <0.05 and the fold change in gene expression was at least 4-fold. Functional clustering for *A. thaliana* was obtained from DAVID (Huang da *et al.* 2009). If a functional cluster had a modified Fisher’s exact test, *P* < 0.05, then those clusters were considered statistically significant and used for further analysis. To obtain the functional clustering for *B. depauperata* and make the data comparable to *A. thaliana*, the *B. depauperata* gene ids were converted to equivalent *A. thaliana* gene ids by using Mercator. Mercator would output a file mapping with *A. thaliana* gene ids that were then inputted in DAVID to obtain functional clustering. The full list of gene ontology (GO) category enrichment for both DE and AS is provided in **Supporting Information—Tables S1** and **S2**.

RT–PCR was used to validate AS events. Briefly, heat stress experiments were replicated and plant leaf tissue was frozen in liquid nitrogen. Total RNA was extracted from frozen homogenized leaf tissue for control and heat-stressed plants (B/HS and A/HS) from both species using the RNeasy Plant Mini Kit. cDNA was generated using the Invitrogen SuperScript IV Reverse Transcriptase synthesis kit. Approximately 100 ng of RNA was used in each SuperScript reaction using the standard protocol. Invitrogen Platinum PCR SuperMix was used for the RT–PCR reactions using standard protocols. The primers used in this analysis are as follows: *A. thaliana* FBS1 (F-box protein S1, At1G61340) Forward primer: CAGCTTTTGTTCATTTCCCAC, Reverse: GACCTATCCAAACCAAAACCC; *A. thaliana* Hsp60.2 (At2G33210) Forward primer: CCAGATTGGAAGCAGGCTCA, Reverseprimer:CACAACTGCTTCCGTCGTTG;*A.thaliana* Hsp21 (At4G27670) Forward: ACTCTCATTTGCTGCATCGGC, Reverse:TGGACATCGATGACTTTGCG;*B.depauperata* HSF4AForward:CCACCTTTCCTCACCAAAAC,Reverse: CCTTCCTCTCTCACAACAAC; *B. depauperata* SR45 (arginine/serine-rich protein) Forward: AGCGTCAGTTCTGGGAGTC, Reverse:CTTCTGGGACTTGGTGAACT;*B.depauperata* Hsp21 Forward: ACTCTCATTTGCTGCATCGGC, Reverse: TGGACATCGATGACTTTGCG.

## Results

### Heat stress induces species-specific patterns of AS

To examine the patterns of AS in *A. thaliana* and *B. depauperata* in response to basal and acquired heat stress, we generated and sequenced control and heat-stressed RNA-seq libraries. The sequence data were then analysed for AS and DE patterns. Using TopHat (Trapnell *et al.* 2009), 82909 SJs were identified in *A. thaliana* control tissue (Table 1). The number of control SJs found here is comparable to the number of SJs previously identified in *A*. *thaliana* (TAIR). Our data indicate that the total number of SJs is affected by heat stress in both species (Table 1). However, in *A. thaliana*, the number of SJs increases after exposure to HS, while in *B. depauperata*, the number of SJs decreased after heat stress. Following a basal heat stress (B/HS) at 38 °C, the number of SJs increased to 86523 in *A. thaliana*. The number of SJs increased again to 95281 after an acquired heat stress (A/HS) at 43 °C (Table 1). In contrast to *A. thaliana*, the number of *B. depauperata* SJs decreased under heat stress. *Boechera depauperata* SJs decreased from 98153 to 82884 following B/HS, and continued to decrease to 79740 after A/HS (Table 1).

**Table 1.** Number of SJs.

	*A. thaliana*	*B. depauperata*
Control	82909	98153
Basal HS	86523	82884
Acquired HS	95281	79740

To gain a detailed understanding of the role of AS during HS, we examined specific types of AS events using DiffSplice (Hu *et al.* 2013). This is a tool for discovering and quantifying AS variants present in an RNA-seq data set, without relying on annotated transcriptome or predetermined splice pattern. These AS events include ES, IR and alternative 3' or 5' splicing (A3'SS/A5'SS). Examination of AS events in each species following heat stress revealed further differences between *A. thaliana* and *B. depauperata* (Table 2). We found 5762 genes in the *A. thaliana* control tissue that had AS events. As was seen in the total number of SJs (Table 1), the number of the *A. thaliana* AS events increased after HS (Table 2). In *A. thaliana*, the total number of AS events (ES, IR and A3'SS/A5'SS) increased after B/HS (6535), and following A/HS (7396) (Table 2). The most common splicing event in *A. thaliana* under all conditions (control and heat stress) was A3'SS/A5'SS (Table 2). Notably, while A3'SS/5'SS was the most frequent splicing event in all *A. thaliana* samples, this type of AS event does not appear to be sensitive to heat stress (i.e. the number of these events was not changed by heat treatment). During control conditions, 96 % (5532 out of 5762) of AS events were A3'SS/A5'SS. However, this percentage deceased after A/HS to ~69 %. This decrease in *A. thaliana* was not due to changes in the number of A3'SS/A5'SS events but rather it was due to increases in the other AS events. The number of IR events, which were quite low (128) in control samples of *A. thaliana*, increased greatly with heat stress: 1576 events in B/HS and 2079 events in A/HS. In *A. thaliana*, ES events were low in control samples and were most frequent after exposure to B/HS.

**Table 2.** Number of AS events in *A. thaliana* and *B. depauperata*. The symbol *indicates value is statistically different from control at the *P* < 0.001 level based on a *t*-test.

	*A. thaliana*				*B. depauperata*			
	Total AS	ES	IR	A3′SS/A5′SS	Total AS	ES	IR	A3′SS/A5′SS
Control	5762	102	128	5532	7911	3464	2487	1960
Basal HS	6535*	217*	1576*	4742	7641	3539	2346	1756
Acquired HS	7396*	146*	2079*	5171	7453	3555	2275	1623

Heat stress has a significant impact on AS events in both species. However, the data reveal considerable differences in how HS affected AS in *A. thaliana* and *B. depauperata*. We found 7911 AS events in *B. depauperata* control tissue, and while the total number of AS events decreased in *B. depauperata* after exposure to heat stress, this reduction was not statistically significant (Table 2). When each type of AS event was analysed individually, we see that HS does not significantly impact the number of AS events in *B. depauperata*. Another major difference between *B. depauperata* and *A. thaliana* was that under all conditions (control, B/HS and A/HS) ES events were always the most frequent in *B. depauperata*, followed by IR, then A3'SS/A5'SS (Table 2). In *A. thaliana* ES events were the least frequent (Table 2).

### Gene ontology analysis of genes undergoing AS reveals species-specific patterns

**Figure 1.**
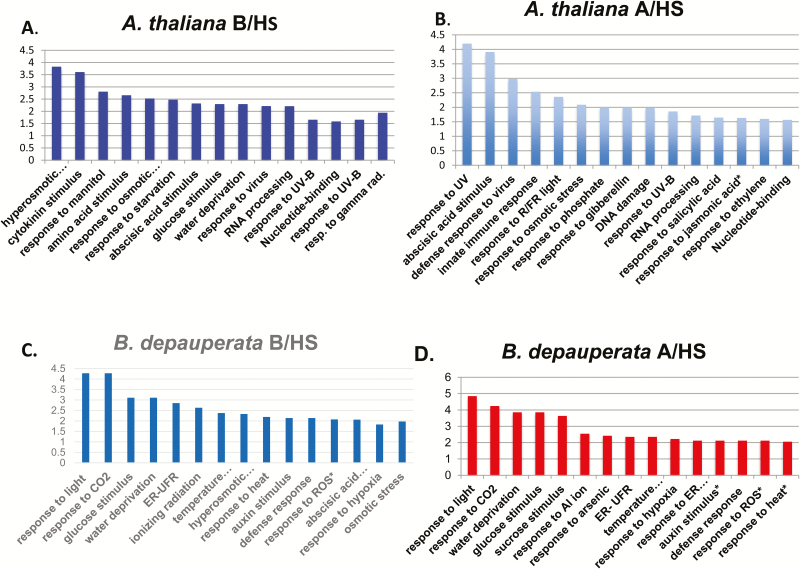
Functional enrichment of genes involved in AS events. Fold enrichment (Log10 (1/*P*-value)) of functional clustering of genes that underwent AS. The functional clustering was obtained from DAVID. Gene ontology terms were included if they had a FDR < 0.01 and a *P*-value < 0.05. Data from basal heat stress are in blue, acquired heat stress in red. (A) *A. thaliana* basal HS; (B) *A. thaliana* acquired HS; (C) *B. depauperata* basal HS; (D) *B. depauperata* acquired HS. All scales are Log10 (1/*P*-value). Categories that are also significantly enriched in DE are indicated by a *.

**Figure 2.**
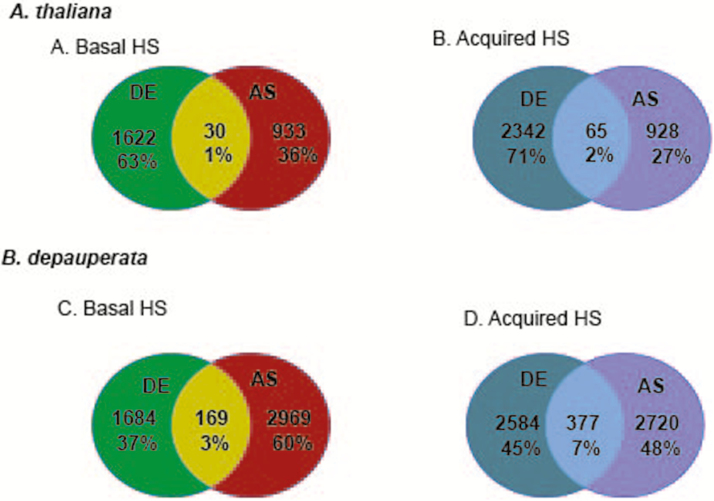
Analysis of AS and HS events. The Venn diagrams indicate the number of genes with AS or DE events, and in the intersection, the number of genes with both AS and DE. (A) *A. thaliana* basal HS. (B) *A. thaliana* acquired HS. (C) *B. depauperata* basal HS. (D) *B. depauperata* acquired HS.

Using the program DAVID (Huang da *et al.* 2009) we examined the enrichment of GO categories in the genes undergoing AS during BHS and AHS in both species. The AS GO category enrichment patterns varied between B/HS and A/HS and were also quite different in *A. thaliana* and *B. depauperata* (Fig. 1). In *A. thaliana* only four GO categories were found to be enriched in AS in both the B/HS and A/HS (response to osmotic stress, RNA processing, response to UV-B and nucleotide binding). However, most AS GO categories were shared between *B. depauperata* B/HS and A/HS treatments. More importantly, our analysis revealed that the functional categories that undergo AS were largely non-overlapping with those that undergo DE. In both species, the vast majority of GO categories that were found to be enriched in AS were not differentially expressed (DE). To further understand the differences between DE and AS during heat stress, we examined the individual genes that underwent AS, DE and those that underwent AS and DE. We found that for both species the individual genes that undergo AS in response to HS are distinct from those that undergo DE (Fig. 2). In *A*. *thaliana* after B/HS, only 1 % of genes are both AS and DE. Similarly, after A/HS, only 2 % of genes are both AS and DE. In *B. depauperata* after B/HS 169 genes are both AS and DE but after A/HS the number of genes that undergo AS and DE increases to 377. While these percentages are slightly higher than that seen for *A. thaliana*, the patterns are largely the same. Genes either undergo AS or DE but not both.

**Figure 3.**
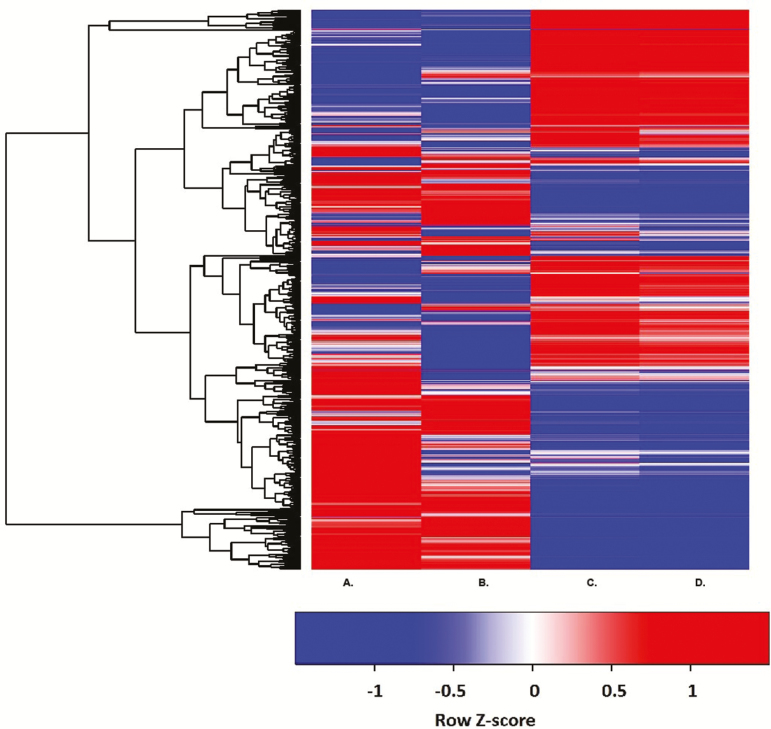
Heatmap of genes that were AS and DE in both *A. thaliana* and *B. depauperata*. The blue lines represent the genes that are down-regulated and the red lines represent the genes that are up-regulated. The values are normalized to common *z*-score to make the plot readable. Each row corresponds to the same gene. Genes are hierarchically clustered. (A) *A. thaliana* basal HS. (B) *A. thaliana* acquired HS. (C) *B. depauperata* basal HS. (D) *B. depauperata* acquired HS.

**Figure 4.**
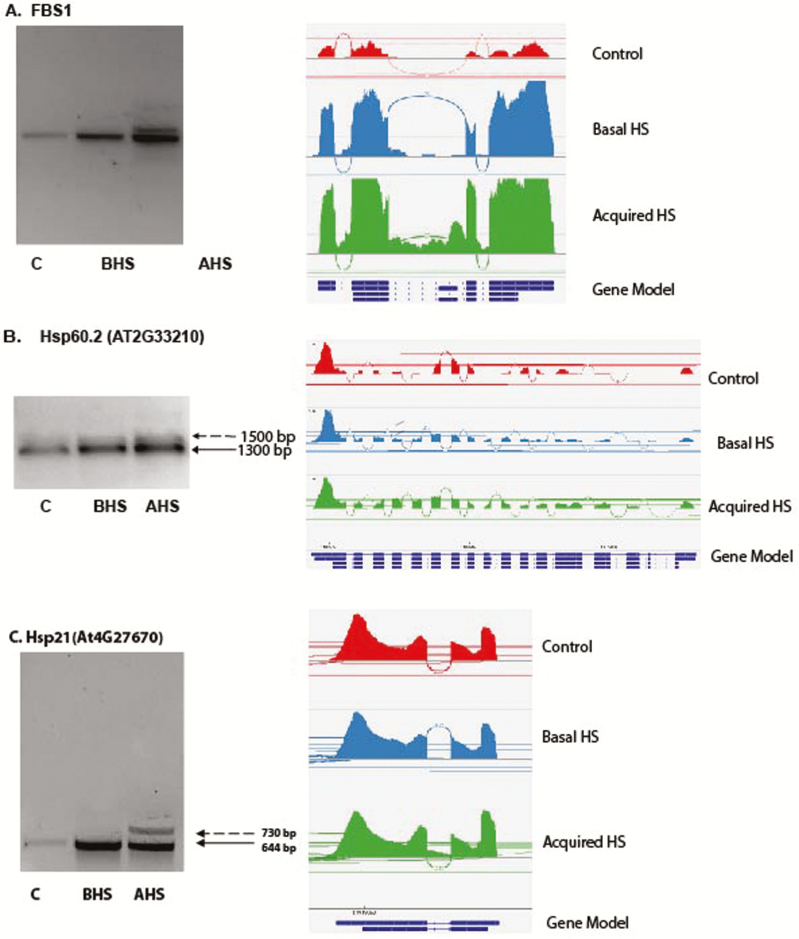

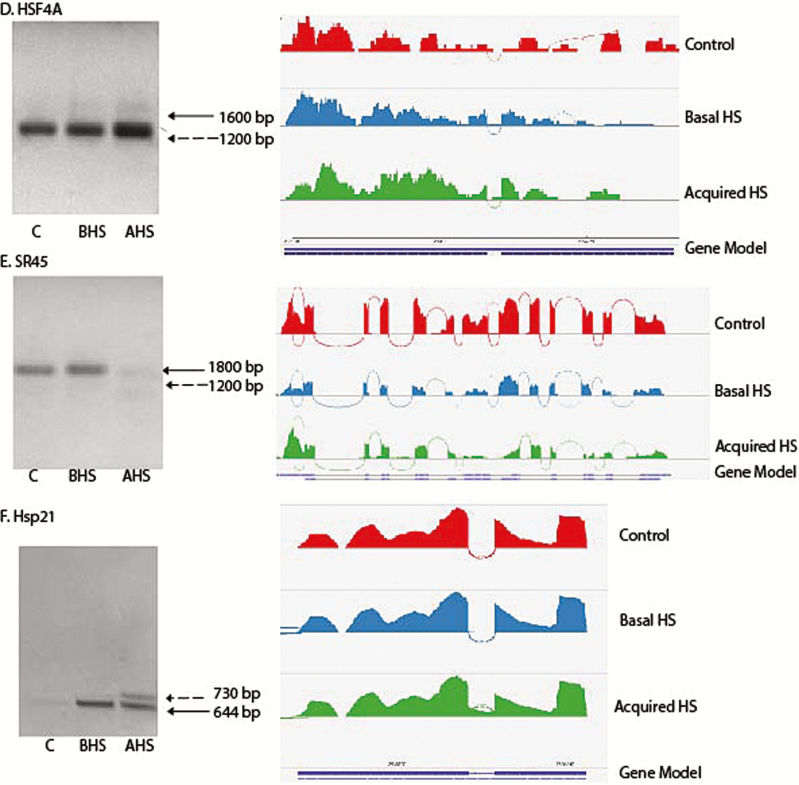
Confirmation of AS events with RT–PCR. The representative AS events in six stress-responsive genes validated by RT–PCR and visualized by IGV browser. In the RT–PCR validation, the dotted arrow indicates the alternative splice form. The regular arrow indicates normal splicing. In the IGV visualization, the exon-intron structure of each gene is given at the bottom of each panel. The sashimi plots obtained from IGV show the AS taking place in each gene. If the sashimi plots show more reads between the expected SJ it suggests that the genes have IR and if the expected SJs are being skipped it suggest that the gens have ES. (A) *A. thaliana* FBS1 (F-Box Protein S1, At1G61340). (B) *A. thaliana* Hsp60.2 (At2G33210). (C) *A. thaliana* Hsp21 (At4G27670). (D) *B. depauperata* HSF4A. (E) *B. depauperata* SR45 (arginine/serine-rich protein). (F) *B. depauperata* Hsp21.Figure 4. Continued.

To gain a deeper understanding of the few genes that did undergo both AS and DE, we examined the individual genes in each species that underwent both. A heatmap was used to compare the expression profile and AS patterns in *A. thaliana* and *B. depauperata* (Fig. 4). *Boechera depauperata* genes were annotated against the *A*. thaliana genome to facilitate comparisons across species. In *A. thaliana* genes that were AS and DE expressed after BHS had similar expression patterns after AHS. However, the *B. depauperta* homologs of those genes did not share the same expression patterns (Fig. 3). This again demonstrates the clear species differences in the AS response to heat stress.

### Validation of AS events

RT–PCR was used to experimentally validate AS events identified in RNA-seq analyses. From the DiffSplice results, we chose genes that were identified as undergoing AS with FDR < 0.01 and had a significant DE *P* < 0.05 when compared to control (22 °C) samples. Alternative splicing events were confirmed for three *A. thaliana* genes: (i) F-box Stress induced 1, *FBS1* (AT1G63140); (ii) Heat shock protein 60-2, *HSP60-2* (At2G33210); (iii) Heat shock protein 21, *HSP21* (At4G27670). In A. *thaliana FBS1*, an F-box protein was found to have IR after A/HS (Fig. 4A). *HSP60-2*, a member of the well-studied HSP60 family, was shown to have an IR event (Fig. 4B). HSP21 protein had IR in *A*. *thaliana* after A/HS (Fig. 4C).

*Boechera depauperata* AS events were also confirmed for three genes: (i) *SR45*, serine/arginine-rich protein 45 (*AT1G16610*); (ii) *HSF4A*, heat shock transcription factor A4A (AT4G18880); (iii) *HSP21* (At4G27670). *SR45* is a member of the highly conserved family of serine/arginine-rich (SR) proteins. These proteins have key roles in pre-mRNA splicing and other aspects of RNA metabolism. The gene encoding SR45 displayed IR in *B*. *depauperata* after A/HS treatment (Fig. 4D)*. HSF4A*, a member of Heat Stress Transcription Factor (Hsf) family (Scharf *et al.* 2012), was also found to have IR in *B*. *depauperata* after A/HS (Fig. 4E). Finally, we confirmed that *HSP21* has an IR event in *B. depauperata* (Fig. 4F) as in *A. thaliana* (Fig. 4C).

## Discussion

The sessile nature of plants makes the evolution of tolerance to stress of primary importance for species survival. The well-studied plant heat stress response is known to involve complex changes in gene expression that, in turn, promote increased thermotolerance (Larkindale *et al.* 2005; Larkindale and Vierling 2008; Mittler *et al.* 2012; Qu *et al.* 2013). We examined the patterns of AS during heat stress in *A. thaliana* and *B. depauperata*, two related species with different levels of tolerance to heat stress. Our studies show that heat stress strongly affects AS. However, the nature impact of HS on AS is different in the two species. In that the relationships between heat stress and the levels and types of AS were significantly different in each of our study species.

Both *A. thaliana* and *B. depauperata* undergo AS during heat stress. However, the differences in AS during HS in *A. thaliana* and *B. depauperata* were evident when the numbers and types of splicing events were compared (Table 2). It is notable that IR was low in *A. thaliana* control tissue, but significantly higher in heat-stressed tissue. In contrast, a different pattern was seen in the more thermally tolerant *B. depauperata* (Table 2). *Boechera depauperata* had high IR levels in control tissue, which decreased during heat stress. One possible explanation is that the presence of IR splice variants was important for thermotolerance and that the high levels of IR variants present in *B. depauperata* are related to higher thermotolerance. A second major difference between *A. thaliana* and *B. depauperata* is the high number of A3′SS/A5′SS events in *A. thaliana* relative to *B. depauperata*. It is possible that the lack of a *B. depauperata* genome negatively impacted the ability to identify splice sites. The 3'/5' splice sites would be expected to evolve much more quickly than the intron/exon boundary sites and it is possible that the use of the transcriptome and *B. stricta* genome hampered our ability to identify these sites. However, since the fact that we obtained the number of SJs and the same SJs with both the *B. depauperata* transcriptome and the *B. stricta* genome gives confidence in our ability to identify AS sites. Even if we underestimated the number of A3'SS/A5'SS events in *B. depauperata*, the completeness of the *B. depauperata* transcriptome and the closeness of the *B. stricta* genome indicates that we accurately annotated *B. depauperata* genes.

A recent study of AS in *A. thaliana* under salt stress (Ding *et al.* 2014) reported that an increase in the number of IR events with salt stress. A study of AS in the moss *P. patens* reported that HS induced an increase in IR events in genes related to protein folding but resulted in a decrease in IR events in genes related to chloroplasts and RNA splicing (Chang *et al.* 2014). These findings as well as those presented here support the conclusion that AS is not a by-product of stress, but is instead, a regulated component of the stress response. Our findings that IR is sensitive to abiotic stress in *A. thaliana* are consistent with these previous studies. However, here we identified fewer IR events in *A. thaliana* control tissues than that found in previous AS studies. Marquez *et al.* (2012) stated that many of the IR events that they identified had low read depth and are not well represented in assembled transcripts. We used stringent criteria for IR events and this could explain the lower number of IR events identified. Despite these differences compared to previous studies the fact that we used the same criteria for both *A. thaliana* and *B. depauperata* and found differences across these species makes our findings significant.

Abiotic stress is not the only influence on patterns of AS events. One other factor that may play a role is that *B. depauperata* is a polyploid. It has been hypothesized that autotetraploidization can alter the transcriptome and modulate AS (Saminathan *et al.* 2015). Alternative splicing patterns have been studied among natural and synthetic polyploids of *Brassica napus*. Two independently synthesized lines showed parallel loss of AS events after polyploidy (Zhou *et al.* 2011). Alternative splicing divergence between duplicated genes may have contributed to gene functional evolution and led to the preservation of some duplicated genes (Zhang *et al.* 2010). The AS patterns of WDREB2 transcripts have been compared among wheat species with different ploidy levels (Terashima and Takumi 2009). Alternative splicing produced functional and non-functional forms of the WDREB2 transcripts and the levels of the non-functional form decreased during drought stress in the diploid progenitors. In contrast, the non-functional form did not decrease in hexaploid wheat lines, thus indicating that allopolyploidization inhibited efficient AS of WDREB2 transcripts (Terashima and Takumi 2009). Therefore, the differences in patterns of AS in *A. thaliana* and *B. depauperata* may be related to the fact that *B. depauperata* is a polyploid. Further analysis of AS during HS in closely related species that vary by ploidy level would help to further evaluate the impact ploidy has on AS.

It has been hypothesized that increased AS could be a result of inaccurate splicing, which in turn could weaken the function of the genes by decreasing the number of corresponding transcripts (Ding *et al.* 2014). It has also been proposed that an increase of AS comes from splicing errors and that stress causes degradation of the splicing machinery (Ling *et al.* 2017). Here we show that some heat shock genes, i.e., *HSP90*, *HSP60*, showed increased ES or alternative 5'SS/3'SS under increased heat stress in both the plants. These transcripts may produce non-functional proteins, but further analyses are required to determine if these AS events significantly alter their *in vivo* function(s).

If AS is a by-product of stress and the splicing machinery is degraded during such periods, we would expect to see a random distribution of AS. Yet, genes associated with stress response and those that are involved in abiotic stimulus are alternatively spliced (Fig. 1). With the production of large amounts of these stress-inducible pre-mRNAs, cells would immediately need to recruit significant amounts of splicing and other factors necessary for post-transcriptional processing. This imposes a huge burden on the splicing machinery, and as a result, a significant portion of these transcripts fails to be processed adequately when splicing machinery is compromised (Ling *et al.* 2017). The analysis of GO categories of the AS genes in both the species (Fig. 1) revealed these categories differed between the two species. Interestingly, in *A. thaliana* RNA processing and nucleotide binding underwent AS under both B/HS and A/HS treatments but these categories were not enriched in the *B. depauperata*.

Another interesting difference between the two species is that genes involved in response to light stimulus undergo AS in *B. depauperata*, but not in *A. thaliana*. Previous research has shown that *B. depauperata* is both more tolerant to high light and combined high light and high heat stress than is *A. thaliana*. *Boechera depauperata* can also maintain photosynthesis at higher temperatures (Gallas and Waters 2015; Halter *et al.* 2017). While the genes associated with photosystem II and photosystem repair did not have enough AS change to be included in Fig. 1 analysis shows that these functional classes do have AS events in *B. depauperata* but not in *A. thaliana* (no fold change in *A. thaliana* and fold change of 0.8 to 1.8 in *B. depauperata*). This suggests that AS is playing a role in the higher stress tolerance of *B. depauperata*.

When our different analyses are examined together, it is clear that significant differences exist in the *A. thaliana* AS response to B/HS and to A/HS. Our finding that *A. thaliana* AS patterns during B/HS are distinct from that of A/HS is consistent with previously published work on *A. thaliana* which indicated that while a number of genes and pathways are shared between B/HS and A/HS in this species there are large differences in the specific genes that are expressed under these different types of heat stress. Our finding of few differences between the B/HS and A/HS AS responses in *B. depauperata* is intriguing and is worthy of future study.

## Conclusions

Our analysis reveals extensive AS in response to heat stress in both *A. thaliana* and *B. depauperata*. Previous studies have shown that *B. depauperata* is highly thermotolerant. One goal of this study was to determine if the patterns of AS differ between these related species. Although it is clear that AS is found in both species, the level and types of AS events differ markedly. Further, there are considerable differences between the genes that undergo AS in the two species. One pattern that is conserved across species is that the genes undergoing AS are mostly distinct from those undergoing DE. This provides further evidence that AS is part of the larger programmed response to heat stress. Future studies will include examining the role of specific AS events on gene/protein function and their impact on organismal thermotolerance.

## Accession Numbers

The raw sequencing reads can be found at the NCBI GEO database with the following accession number GSE107820.

## Sources of Funding

This project was supported by a grant from the National Science Foundation (IOS-0920611) and by a CSUPERB Research Development Award to E.R.W.

## Contributions by the Authors

S.K. analysed the data and assisted in preparing the manuscript; G.H. performed the heat stress experiments; T.R. assisted in data analysis and in preparation of the manuscript; E.R.W. planned and organized the experiments, oversaw data production and analysis, prepared the manuscript, and secured the necessary funding.

## Conflict of Interest

None declared.

## Supplementary Material

Supplementary Table 1

Supplementary Table 2
